# Unexpected Thrombocytopenia in a Parturient With Evans Syndrome Complicated by COVID-19 Infection

**DOI:** 10.7759/cureus.27409

**Published:** 2022-07-28

**Authors:** Mohamed Fayed, Shuchi Jain, Nyla Leonardi, Joshua Younger

**Affiliations:** 1 Anesthesiology, Pain Management and Perioperative Medicine, Henry Ford Health System, Detroit, USA; 2 Anesthesiology and Critical Care, Cleveland Clinic, Cleveland, USA; 3 Anesthesiology and Perioperative Medicine, Wayne State University, Detroit, USA; 4 Anaesthesiology, Henry Ford Health System, Detroit, USA

**Keywords:** mycophenolate mofetile, intravenous immunoglobulins (ivig), s: anemia, auto-immune, covid 19, thrombocytopenia, acute pulmonary embolism, covid associated thrombocytopenia, labor epidural analgesia, evans syndrome

## Abstract

We report the case of a 23-year-old parturient who received epidural analgesia and was subsequently diagnosed with Evans syndrome (ES). The diagnosis was made after a complete blood count (CBC) resulted in severe anemia and a platelet count of less than 10K/µL. To further complicate this case, the patient developed post-delivery pleuritic chest pain and pulmonary emboli (PE), and a chest computed tomography (CT) scan showed bilateral ground-glass lung opacities. This prompted a COVID-19 testing and ultimately confirmed infection.

## Introduction

Evans syndrome (ES) is a rare autoimmune hematological disease defined as the co-occurrence of two or more immune cytopenias, commonly affecting red blood cells and platelets, with resultant autoimmune hemolytic anemia (AIHA) and immune thrombocytopenia (ITP) [1]. Less often, patients also have autoimmune neutropenia [2]. The diagnosis of ES is a diagnosis of exclusion. Once other causes of thrombocytopenia are ruled out, and a positive direct antiglobulin test (DAT) is present, ES can be diagnosed. ES is more challenging to treat than isolated warm AIHA because it is less responsive to standard therapies.

Additionally, patients with ES have more frequent relapses and higher mortality. The first line treatment is glucocorticoids. Other treatment modalities are often combined with intravenous immune globulin (IVIG) and can include rituximab or mycophenolate mofetil. These second-line agents are usually employed when the platelet count is less than 20K/µL [3].

The development of ES in pregnancy is scarce, and only a few cases have been reported [4]. Given the rarity, there are no well-defined treatment protocols [5,6]. In the recent literature, COVID 19 has been established as a trigger for ES [7,8]. In another case report, the AIHA antibodies can be passed to the fetus and result in anemia [9]. Another patient developed disseminated tuberculosis infection with steroids treatment [10]. 

Here we present the case of a parturient who received neuraxial anesthesia in active labor and was subsequently found to have severe thrombocytopenia due to ES. In this case, the patient's epidural catheter was placed and removed without complication or knowledge of the thrombocytopenia. The patient's postpartum state was complicated by a pulmonary embolism (PE) and COVID-19.

## Case presentation

A 23-year-old 38 weeks pregnant presented to the labor and delivery ward in active labor. She reported a history of iron deficiency anemia and mild asthma. Physical exam findings were unremarkable. Her most recent complete blood count (CBC) was from three years prior and included a platelet count of 223 K/µL (table 1).

**Table 1:** Patient's complete blood count during admission and 3 years prior. WBC: white blood cell, RBC: red blood cell, HCT: hematocrit, MCV: mean corpuscular volume, K: thousand, M: million.

	Reference Range & Units	3 years prior	1^st ^sample	Repeat sample	3 days later	5 days later
WBC Count	*3.8 - 10.6 * *K/uL*	13.6	9.9	10.5	17.8	13.7
RBC Count	*4.1 - 5.5 * *M/uL*	4.51	2.96	2.31	3.15	3.44
Hemoglobin	*12- 15 * *g/dL*	12.5	7.1	5.6	8.3	9.1
HCT	36 - 46 %	37.6	22.1	17.7	26.0	28.2
MCV	80 - 100 fl	83.4	74.7	76.5	82.4	82.1
Platelet Count	*150 – 450 * *K/uL*	223	<10	<10	55	92

A labor epidural was placed without incident before laboratory results. An experienced resident inserted the epidural catheter, which was straightforward. Laboratory workup showed CBC had a platelet count of less than 10K/µL and hemoglobin of 5.6g/dl. These abnormal results were thought to be erroneous, and a second sample was sent. The patient had an uncomplicated vaginal delivery with an estimated blood loss of 400mL, and the epidural catheter was removed. Repeat CBC confirmed a platelet count of <10K/µL and hemoglobin of 5.9g/dl. The blood film showed microcytic hypochromic anemia with anisocytosis and polychromasia. Given the concern for a potential epidural hematoma, the patient was monitored for 48h with frequent neurologic examinations and assessments for developing spinal-epidural hematoma (SEH). She continued to have intact and unaffected sensory and motor function in the bilateral lower extremities and normal bladder and bowel function. Other laboratory results revealed an elevated lactate dehydrogenase, normal coagulation parameters, and positive DAT. Tests for hepatitis C and human immunodeficiency virus were negative. Urine analysis did not show evidence of proteinuria. Bone marrow biopsy did not show any evidence of neoplastic cells. Computed tomography (CT) of the abdomen did not show evidence of splenic laceration or splenomegaly.

The patient received two units of packed red blood cells and one pooled platelet unit. The patient was prescribed oral dexamethasone 40mg daily for four days. Her hemoglobin and platelet count improved with hemoglobin of 8.3g/dl and platelet count of 55K/µL. She was discharged on 60mg of oral prednisone daily. During outpatient monitoring, her platelet count once again fell below 10K/µL. She was readmitted twice, once due to hematemesis and again due to bruising and low platelet count demonstrated in an outpatient laboratory testing. She was treated with another dose of dexamethasone and intravenous immunoglobulin.

**Figure 1:**
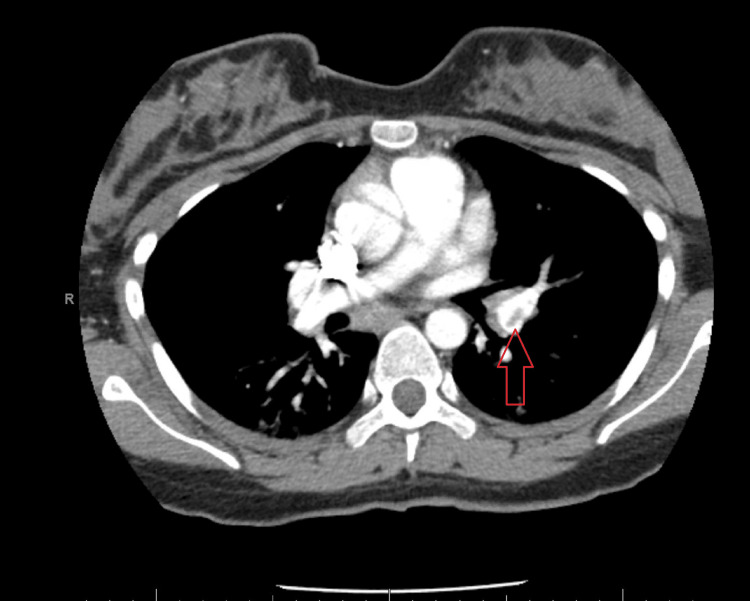
Patient's CT pulmonary angiogram. Red arrow: clot in the left pulmonary artery.

**Figure 2:**
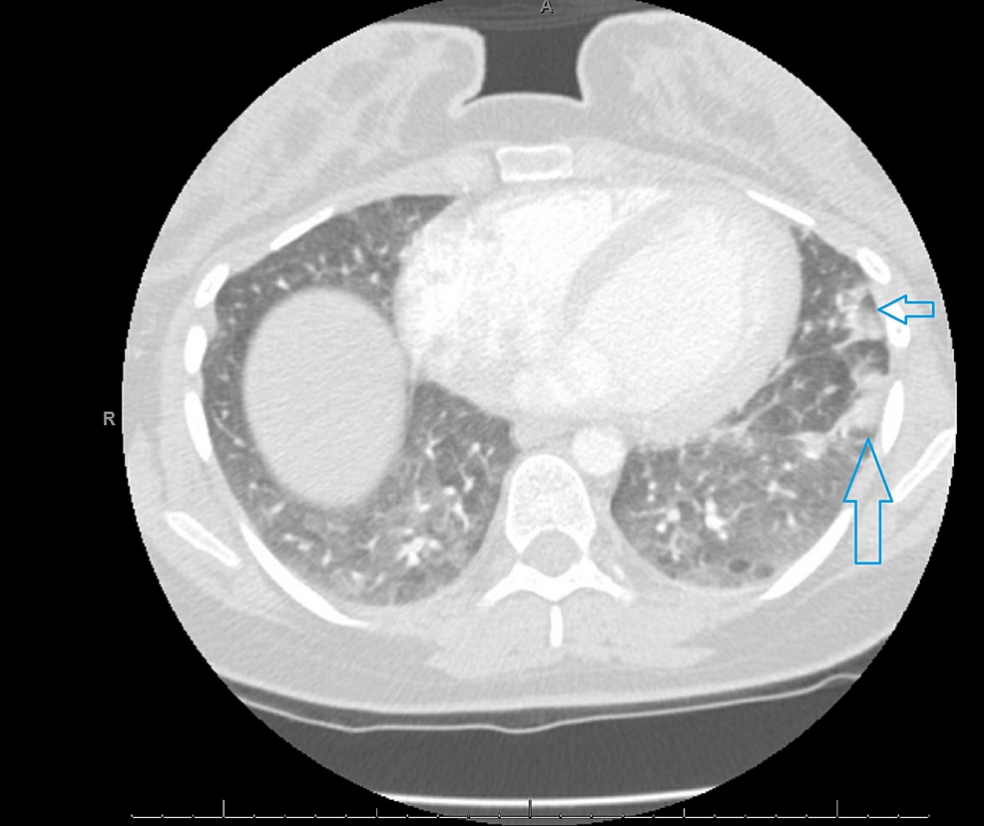
Patient's CT chest, lung window. Blue arrow: pneumonic patch

Four weeks post-delivery, she presented to the emergency department reporting pleuritic chest pain and shortness of breath. A CT pulmonary angiography showed PE in the segmental branches of the lingula and the inferior lobar artery extending into the segmental branches of the lower lobe (figure 1). There were bilateral diffuse ground-glass opacities, which prompted a COVID-19 test (figure 2).

Test results came back positive. Her calculated sequential organ failure score was 2, and the platelet count of less than 100K/µL contributed to this score. Four days later, the patient was stable and discharged home to self-quarantine.

## Discussion

Administering neuraxial epidurals in obstetric patients can be complicated by a new diagnosis of a rare hematologic condition such as ES. When our patient arrived at the hospital in labor, her benign medical history and physical exam made her a good candidate for a labor epidural upon request. After labor epidural placement and parturition, the platelet count was less than 10 K/µL. The positive DAT and the presence of thrombocytopenia and anemia confirmed the patient's diagnosis of ES. Although she did not experience any adverse outcomes when the labor epidural was inserted, her treatment course was complicated by pulmonary embolism and a positive COVID-19 diagnosis.

As the diagnosis of ES is a diagnosis of exclusion, several other diagnoses needed to be ruled out. These diagnoses range from pregnancy specific to general causes of thrombocytopenia: Preeclampsia with severe features, Hemolysis, Elevated Liver enzymes, Low Platelet (HELLP) syndrome, and disseminated intravascular coagulopathy or even regular physiological changes with pregnancy. Our patient did not have a history of hypertension or evidence of proteinuria. She also had normal liver enzymes, a normal coagulation profile, and thrombocytopenia that was too profound to be considered a regular physiologic change. With these findings, the above-mentioned pregnancy-related conditions were reasonably excluded. Non-pregnancy-specific causes of thrombocytopenia are TTP, HUS, hypersplenism, or malignancy. The patient's renal functions were normal; her blood smear did not show fragmented cells, and her basement membrane biopsy ruled out malignancy. This extensive evaluation suggested an alternative diagnosis, and when paired with the positive DAT, anemia, and thrombocytopenia helped confirm the diagnosis of ES. 

Our patient was readmitted in the postpartum state with pleuritic chest pain; she was confirmed to have both PE and COVID-19 infection. Given the novelty of COVID-19, only a few case reports and two studies demonstrate that COVID-19 causes a hypercoagulable state, increasing the risk of venous thromboembolism (VTE) [11,12]. The patient's CT scan showed patchy pneumonia, and possible immunosuppression with steroids was protective against respiratory deterioration and respiratory failure [13]. Despite data suggesting a correlation of SOFA score with COVID-19 pneumonia, thrombocytopenia from ES can be a confounding factor, as in this case [14]. Assessing the risk and benefits of administering methergine in these cases is worth noting to avoid pulmonary artery vasoconstriction and myocardial injury [15]. The pathophysiology of ES remains poorly understood, but thrombosis is a known complication of ITP and AIHA [16]. 

In obstetric patients with severe thrombocytopenia, neuraxial anesthesia is contraindicated due to the increased risk of SEH [17]. Per Lee et al., the estimated risk of SEH in patients with thrombocytopenia ranging from 0 to 49 K/µL is 11% [18]. However, the current American Society of Anesthesiologists and Society for Obstetric Anesthesia and Perinatology guidelines state that routine platelet testing is unnecessary for healthy obstetric patients [19]. Given our patient's benign medical history and physical exam, it is reasonable that a labor epidural was offered and placed before laboratory results were available. The subsequent discovery of severe thrombocytopenia with platelets <10K/uL challenges these ASA and SOAP guidelines. It makes one reconsider the previously stated recommendations and emphasizes the need for a thorough review of a patient's hematologic medical history. In this era of the COVID-19 pandemic and its uncertainty, hypervigilance and admission laboratory workup should be present before neuraxial epidural treatment.

## Conclusions

ES is an autoimmune disease that is mainly associated with anemia and thrombocytopenia. We presented a case of ES that was diagnosed after epidural analgesia for labor pain. The patient needed treatment with steroids, IVIG, packed red blood cells, and platelet transfusion. This was complicated by PE and COVID-19 pneumonia, which made treatment more challenging. We hope that this case report will increase awareness of the management of ES and will help clinicians treat this rare disease, especially during pregnancy and delivery. Thrombocytopenia may have been diagnosed and treated before epidural insertion and avoided potential lethal complications.
